# An Unusual Case of Essential Thrombocythemia and Acute Kidney Injury: Case Report and Literature Review

**DOI:** 10.3390/diseases13050162

**Published:** 2025-05-21

**Authors:** Celia Rodríguez Tudero, Alberto Martín Arribas, Patricia Antúnez Plaza, José C. De La Flor, Alexandra Lizarazo Suárez, María Pilar Fraile Gómez

**Affiliations:** 1 Department of Nephrology, Hospital Universitario de Salamanca, 37007 Salamanca, Spain; crodrigueztudero@usal.es (C.R.T.); amartinar@saludcastillayleon.es (A.M.A.); mlizarazo@saludcastillayleon.es (A.L.S.); mpfraile@saludcastillayleon.es (M.P.F.G.); 2 Surgery Doctoral Program, Faculty of Medicine, University of Salamanca, 37007 Salamanca, Spain; 3 Department of Anatomic Pathology, Hospital Universitario de Salamanca, 37007 Salamanca, Spain; pantunez@saludcastillayleon.es; 4 Department of Nephrology, Hospital Central Defense Gomez Ulla, 280467 Madrid, Spain; 5 Health Sciences Doctoral Program, Faculty of Medicine, Alcala University, 28805 Madrid, Spain; 6 Department of Medicine and Medical Specialties, Faculty of Medicine, Alcala University, 28805 Madrid, Spain

**Keywords:** essential thrombocythemia (ET), acute kidney injury (AKI), myeloproliferative neoplasms (MPNs)

## Abstract

Background: Essential thrombocythemia (ET) is a myeloproliferative neoplasm characterized by the uncontrolled proliferation of megakaryocytes and sustained thrombocytosis. Although its impact on renal function is not well established, a few case reports have described glomerular involvement and associated kidney impairment. Case Report: We present the case of a 79-year-old man with ET and stage 3b/A2 chronic kidney disease (CKD), who was admitted with severe acute kidney injury (AKI). This episode was associated with a progressive rise in platelet count, reaching 1,350,000/μL after discontinuation of anagrelide and loop diuretics. Renal biopsy (RB) revealed structural lesions compatible with a myeloproliferative neoplasm, including acute tubular necrosis (ATN), glomerulomegaly, and thrombotic microangiopathy (TMA). Cytoreductive therapy with hydroxyurea and corticosteroids was initiated, resulting in improvement of renal function and achievement of complete hematologic remission. Discussion: During follow-up, a linear correlation was observed between increasing platelet counts and declining renal function, underscoring the need for dynamic therapeutic adjustment and close monitoring to prevent progression to end-stage renal disease (ESRD). Conclusions: This case highlights the importance of nephrological evaluation in patients with ET and supports the role of cytoreductive therapy in managing ET-associated renal complications.

## 1. Introduction

Myeloproliferative neoplasms (MPNs) represent a heterogeneous group of hematologic disorders characterized by the clonal proliferation of hematopoietic stem cells. Among these, essential thrombocythemia (ET) is one of the most frequent subtypes, accounting for approximately 25–30% of chronic MPNs. It has an annual incidence ranging from 1 to 2.5 cases per 100,000 individuals and primarily affects patients between 60 and 70 years of age [1]. ET is defined by a persistent elevation in platelet count due to abnormal megakaryocyte proliferation in the bone marrow, predisposing patients to both thrombotic and hemorrhagic complications [2].

Although the renal implications of ET have been rarely described, emerging evidence suggests a potential association between MPNs and progressive kidney damage [3].

Recent studies have identified a distinctive form of renal involvement termed MPN-related glomerulopathy, characterized by mesangial sclerosis, segmental glomerulosclerosis, chronic thrombotic microangiopathy (TMA), and intraglomerular megakaryocyte infiltration [2].

Patients with MPNs may present with a broad spectrum of renal abnormalities, ranging from mild proteinuria to end-stage renal disease (ESRD). These manifestations are believed to arise from both microvascular thrombotic events and hematopoietic infiltration of the renal parenchyma, leading to structural injury and functional impairment [4]. Specifically, excessive thrombocytosis in ET may contribute to renal dysfunction through endothelial activation, microthrombus formation, and altered glomerular perfusion [5]. Although the pathophysiology remains incompletely understood, renal biopsy findings often resemble those seen in TMA, including basement membrane thickening, mesangial proliferation, and progressive glomerulosclerosis [6]. Furthermore, megakaryocyte accumulation within the renal microvasculature has been implicated in tissue hypoperfusion and ischemia, which may exacerbate renal injury. These biopsy findings support the hypothesis that uncontrolled thrombocytosis may accelerate renal deterioration, underscoring the importance of close monitoring and individualized therapeutic adjustment.

A key element in the pathogenesis of renal involvement in ET is the presence of somatic mutations, particularly Janus kinase 2 (JAK2), most commonly the V617F mutation, where valine is replaced by phenylalanine at codon 617. Additional mutations in the calreticulin (CALR) gene (typically in exon 9) and the myeloproliferative leukemia virus oncogene (MPL), which encodes the thrombopoietin receptor (TPO-R), have also been associated with increased thrombotic risk and endothelial damage—two major contributors to renal dysfunction [7]. Elevated levels of platelet-derived growth factor (PDGF) have also been implicated in the progression of both glomerular and tubulointerstitial damage, further increasing the risk of ESRD in patients with MPNs [8].

Cytoreductive therapy plays a central role in the management of ET by reducing thrombotic risk. However, the selection of cytoreductive agents in patients with concurrent renal dysfunction requires careful consideration. Hydroxyurea is considered the agent of choice in this setting due to its favorable renal safety profile, whereas anagrelide has been associated with a higher incidence of nephrotoxicity [9]. Nevertheless, the impact of cytoreductive therapy on renal outcomes in ET remains insufficiently explored, and further research is needed to define optimal management strategies.

In this context, we describe an unusual case of a patient with long-standing ET and stage G3a/A2 chronic kidney disease (CKD) of unknown etiology, who developed severe acute kidney injury (AKI) following the discontinuation of anagrelide. We explore the temporal relationship between thrombocytosis and worsening renal function, and we emphasize the importance of appropriate cytoreductive management and multidisciplinary nephrology-hematology collaboration to prevent progression to ESRD.

## 2. Case Report

We report the case of a 79-year-old man with a longstanding diagnosis of ET, established more than a decade ago, who had achieved sustained hematologic control with anagrelide at 1 mg/day. His past medical history was notable for non-valvular atrial fibrillation (NVAF), managed with acenocoumarol^®^; benign prostatic hyperplasia; and stage G3a/A2 CKD of unknown etiology. Renal function had remained stable over time, with a baseline serum creatinine of 2.2 mg/dL and a urine albumin-to-creatinine ratio (UACR) of 42 mg/g, in the absence of microhematuria.

Although no prior RB was available to establish a definitive etiology of CKD, it was considered non-specific. Nonetheless, a multifactorial pathogenesis was deemed likely, including chronic loop diuretic use, age-related decline in renal function, and potential nephrotoxic exposures. Notably, ET itself may have played a direct pathogenic role in the development of chronic glomerular injury.

In this context, the patient’s treatment regimen included loop diuretics (furosemide 40 mg/day), low-dose beta-blocker therapy (carvedilol 2.5 mg/day), darbepoetin alfa (80 µg weekly), and oral iron supplementation. He presented to the emergency department with asthenia, progressive functional decline, oliguria, and generalized edema (anasarca) of one week’s duration. Importantly, these symptoms coincided with the recent discontinuation of anagrelide and loop diuretics, which had been stopped seven days prior during an outpatient hematology consultation in response to a rise in serum creatinine (Cr) to 2.8 mg/dL, likely related to recent use of nonsteroidal anti-inflammatory drugs (NSAIDs).

At presentation, the patient was alert and oriented, with a blood pressure of 134/95 mmHg and a heart rate of 125 bpm. He exhibited scrotal edema and bilateral pitting edema extending to the proximal lower extremities. Electrocardiogram (ECG) revealed atrial fibrillation with a rapid ventricular response of 125 bpm. Laboratory tests showed AKI stage 3 according to the 2021 Kidney Disease: Improving Global Outcomes (KDIGO) criteria [10], with serum Cr of 7.2 mg/dL and urea of 262 mg/dL, along with severe hyperkalemia (7.5 mmol/L). Venous blood gas analysis showed mild acidosis (pH 7.29, bicarbonate 20.5 mmol/L, pCO_2_ 45 mmHg).

Initial laboratory evaluation revealed 1,193,000 platelets/μL, 13,830 leukocytes/μL, and normocytic, normochromic anemia, with no elevation in acute-phase reactants. Hyperkalemia management was initiated using 1/6 M sodium bicarbonate, 500 mL of glucose solution with 10 units of regular insulin (Actrapid^®^), and intravenous furosemide (125 mg every 8 h), successfully reducing potassium levels to 5.2 mmol/L.

Imaging studies supported these findings, renal ultrasound showed kidneys of normal size and echotexture without lithiasis. A peripheral blood smear (PBS) confirmed findings consistent with previously diagnosed ET. Additional imaging studies—including chest radiography, renal Doppler ultrasound, transthoracic echocardiography, and fundoscopy—were unremarkable.

Prior to the AKI episode, the patient had stable normocytic, normochromic anemia. Baseline laboratory values showed hemoglobin levels around 10 g/dL, ferritin of 45 ng/mL, and transferrin saturation of 15%. These findings supported the indication for darbepoetin alfa and oral iron supplementation in the context of CKD-related anemia, in accordance with standard nephrology guidelines.

Given the progressive decline in renal function and the presence of proteinuria (0.78 g in a 24-h urine collection), and considering the clinical context, empirical treatment was initiated for suspected acute interstitial nephritis (AIN) of immunoallergic origin, likely secondary to NSAID exposure. The patient received three pulses of intravenous methylprednisolone (250 mg each), followed by a tapering regimen of oral prednisone starting at 1 mg/kg/day.

However, despite depletive therapy with high-dose furosemide (250 mg/day), anasarca, oliguria, and acute pulmonary edema persisted. As a result, the patient required renal replacement therapy (RRT) in the form of intermittent hemodialysis, undergoing a total of four sessions. Because of the hematologic context including the increased bleeding risk associated with ET and the extreme thrombocytosis, a single session of therapeutic thrombocytapheresis was performed prior to initiating dialysis.

To rule out immune-mediated etiologies, the diagnostic workup for AKI was extended with an autoimmune panel that included serum protein electrophoresis (no monoclonal spikes detected), antinuclear antibodies (ANA), anti-neutrophil cytoplasmic antibodies (ANCA), anti–double-stranded DNA (anti-dsDNA), anti–phospholipase A2 receptor (anti-PLA2R), anti–glomerular basement membrane (anti-GBM), anti-Ro, anti-La, anti-streptolysin O (ASLO), serum free light chains, Bence Jones protein in urine, and serologies for hepatotropic viruses and HIV—all of which were negative. Additional laboratory findings are summarized in Table 1.

A renal biopsy (RB) was performed to determine the underlying etiology. Histological analysis revealed the following findings: Light microscopy (LM) revealed 21 glomeruli, two of which were globally sclerotic (9.5%). The glomeruli showed glomerulomegaly with dilation of Bowman’s capsule, resulting in a pseudocystic appearance and mild-to-moderate glomerulocystic changes accompanied by capillary loop retraction. Several glomeruli exhibited microaneurysms with dilated capillary loops and occasional congestion. Discrete mesangial expansion and mild proliferation were observed, with up to seven cells per mesangial axis in some glomeruli. No extracapillary proliferation, fibrinoid necrosis, or double contours were identified. Additionally, no subendothelial or subepithelial deposits were observed on hematoxylin and eosin (H&E), Masson’s trichrome, or periodic acid–Schiff (PAS) staining.

Intraluminal megakaryocytes were detected in glomerular and peritubular capillaries and stained positively for CD61. No microthrombi were observed on H&E, trichrome, or immunohistochemistry (IHC) for CD69. The tubulointerstitial compartment demonstrated a dense mixed inflammatory infiltrate occupying approximately 75% of the interstitial surface, composed predominantly of monocytes, plasma cells, a few epithelioid histiocytes, polymorphonuclear leukocytes, and eosinophils.

In the tubular compartment, changes consistent with ATN were observed, including epithelial desquamation into the tubular lumen, formation of cellular casts with debris, nucleomegaly with prominent nucleoli, and hemosiderin-laden cytoplasm consistent with prior hemorrhage. PAS-positive hyaline casts were noted, without evidence of light chain restriction (kappa/lambda) or amyloid A (AA) deposition. Focal tubulitis was also observed. SV40 staining was negative.

Assessment of chronicity markers revealed minimal changes. No significant interstitial fibrosis or tubular atrophy was identified on Gomori’s trichrome stain. Mild subintimal fibrosis was present in arterial vessels, most notably in one arcuate artery, although assessment was limited by tangential sectioning. Hyalinosis was seen in two arterioles. No thrombi or fibrinoid necrosis were noted.

**Figure 1:**
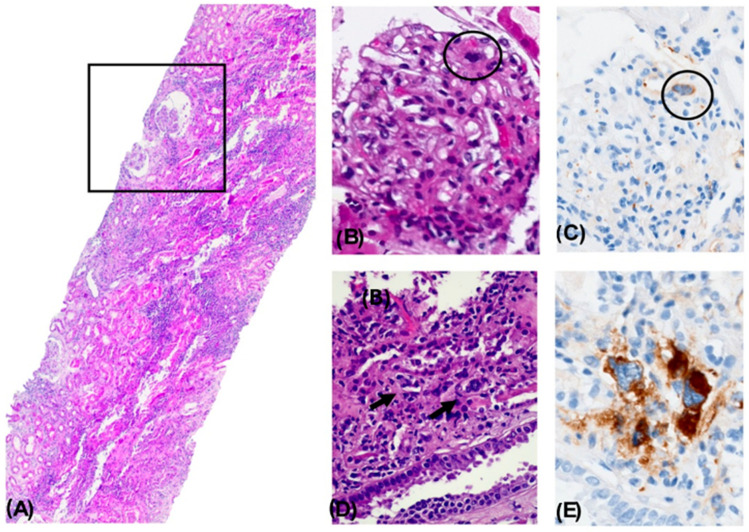
Nephropathy with Histological Changes Associated with a Myeloproliferative Disorder. Panoramic view of a renal biopsy core showing a mild architectural alteration due to increased cellular density and interstitial expansion. A focal pseudocystic change with an enlarged Bowman’s space is observed (square). A dense mixed inflammatory infiltrate is seen in the tubulointerstitial compartment, occupying approximately 75% of the surface, accompanied by acute tubular necrosis. H&E, 4× (**A**). A glomerulus showing capillary lumen obstruction by a cell with a large lobulated nucleus and dense chromatin (circle). H&E, 40× (**B**). Immunohistochemical staining positive for CD61 in an intraluminal megakaryocytic cell. 40× (**C**). Presence of megakaryocytes within the lumens of peritubular capillaries. H&E, 40× (**D**). Immunohistochemical staining positive for CD61, confirming the megakaryocytic lineage. 40× (**E**).

Direct immunofluorescence (DIF) did not reveal any deposition of immunoglobulins or complement components. There was no evidence of kappa or lambda light chain restriction or amyloid deposition. Immunoglobulins were absent as well (Figure 1A–E). Electron microscopy (EM) could not be performed due to insufficient glomerular tissue.

Altogether, the histopathological findings were diagnostic of nephropathy with histologic features associated with a myeloproliferative neoplasm, most consistent with ET. Findings included focal intraluminal hematopoietic cells in glomerular and peritubular capillaries (CD61+), discrete mesangial expansion and mild proliferation, glomerulocystic changes with secondary glomerular retraction, microaneurysm formation, ATN, and extensive interstitial inflammation (75%).

The biopsy findings confirmed renal involvement secondary to ET, with no evidence of AIN as the underlying cause of AKI. Based on these results, cytoreductive therapy was initiated, and the hematologic evaluation was completed with a bone marrow biopsy (BMB). The patient had previously tested negative for the JAK2V617F mutation. Following this, additional molecular testing was performed for CALR and MPL mutations, both of which were also negative.

Histologic analysis of the BMB demonstrated a hypercellular marrow for the patient’s age, with panmyelotic proliferation and numerous large, hyperlobulated megakaryocytes forming dense clusters, including occasional dysplastic forms. A sparse interstitial lymphocytic infiltrate was noted, predominantly composed of CD3+ T cells. There was no increase in the CD34+ blast population. Moderate reticulin fibrosis (grade MF-2) was observed.

**Figure 2:**
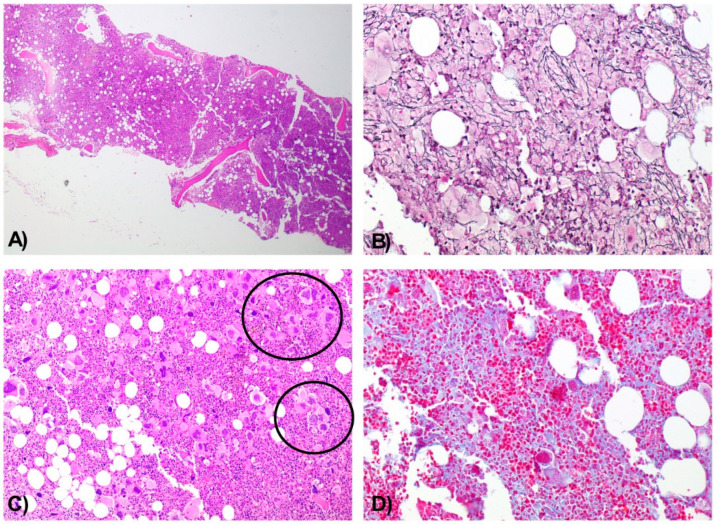
Bone Marrow Biopsy Consistent with a Chronic Myeloproliferative Neoplasm with Moderate Fibrosis. Hypercellular bone marrow for the patient’s age, with panmyelotic proliferation. H&E, 4× (**A**). Moderate reinforcement of the reticulin network (MF2). Wilder, 10× (**B**). Numerous large, hyperlobulated megakaryocytes with a tendency to form dense aggregates (circles) and occasional dysplastic forms. H&E, 10× (**C**). Moderate fibrosis. Masson’s trichrome, 10× (**D**).

Together, these findings supported the diagnosis of a chronic myeloproliferative neoplasm with moderate fibrosis (Figure 2A–D).

Following therapeutic thrombocytapheresis, the platelet count decreased to 691,000/μL. Consequently, a joint decision was made with the Hematology team to initiate cytoreductive therapy with hydroxyurea at a dose of 500 mg daily.

Four weeks after the RB, the patient presented with mild lower limb edema, preserved urine output (~1.5 L/day), and a serum creatinine level of 2.7 mg/dL, with a blood urea nitrogen of 261 mg/dL. There were no signs or symptoms of uremia, and no additional RRT was required.

At the one-month hematology follow-up visit after hospital discharge, the patient showed stable anemia (hemoglobin 9.5–10 g/dL) without signs of clinical decompensation. However, due to a subsequent increase in platelet count to 783,000/μL, the hydroxyurea dose was temporarily increased to 500 mg twice daily, resulting in good hematologic control and tolerance. Renal function exhibited a slight decline in estimated glomerular filtration rate (eGFR), with no significant proteinuria.

Oral anticoagulation with acenocoumarol^®^ was maintained due to the history of NVAF. In parallel, intravenous iron and ESA therapy were introduced to manage anemia. At six months post-hospitalization, renal function remained stable (serum creatinine 2.7 mg/dL, CKD-EPI 20–25 mL/min), hemoglobin was 10.4 g/dL, and ferritin level was 215 ng/mL.

From a hematologic standpoint, platelet counts remained controlled below 600,000/μL with hydroxyurea 500 mg administered every other day. The cytoreductive regimen was titrated according to platelet trends. Darbepoetin alfa (Aranesp^®^) was maintained at 60 µg weekly, aiming to keep hemoglobin levels within the target range of 10–12 g/dL for CKD-related anemia.

At the most recent hematology follow-up, the patient had a platelet count of 489,000/μL while receiving hydroxyurea 500 mg once daily. Given the adequate hematologic response and good treatment tolerance, this dosage was maintained.

## 3. Discussion

This case represents a diagnostically uncommon presentation of ET with direct renal involvement. The histological identification of megakaryocytes within glomerular and peritubular capillaries is a rare and striking finding, suggesting direct infiltration by hematopoietic elements—one of the hallmark features of ET-associated glomerulopathy. Only a limited number of case reports have described such lesions, making this case particularly noteworthy. Moreover, this occurred in the context of AKI superimposed on chronic CKD, with a clear temporal association between rising platelet counts and progressive renal dysfunction. These findings highlight a multifactorial pathogenesis involving neoplastic infiltration, vascular injury, and potential nephrotoxic exposures. This pathophysiological context is exemplified in the following clinical scenario.

**Figure 3:**
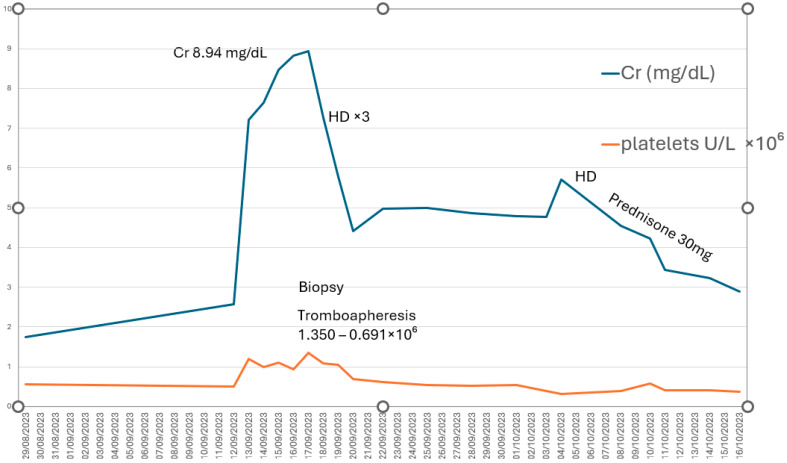
Direct correlation between platelet count (U/L × 10^6^) and renal function (creatinine, mg/dL). HD: hemodialysis.

We report the case of a 79-year-old man with stable ET who developed severe AKI (KDIGO stage 3) requiring RRT with four sessions of hemodialysis, following discontinuation of cytoreductive treatment. The clinical course, histopathologic findings, and—most importantly—the recovery of both renal function and hematologic parameters demonstrate a direct correlation between thrombocytosis and renal deterioration (Figure 3).This supports the hypothesis that markedly elevated platelet counts may play a central role in the pathogenesis of AKI. Thrombocytosis promotes a proinflammatory and prothrombotic state, compromises renal microcirculation, and contributes to both glomerular and tubulointerstitial injury, ultimately resulting in microvascular obstruction and severe interstitial inflammation [7].

This case demonstrates an episode of acute-on-chronic kidney injury, in which chronic histological features such as mesangial sclerosis and interstitial fibrosis coexisted with ATN. This dual pathology reflects the cumulative impact of long-standing MPN-related injury and superimposed acute events contributing to renal deterioration. Corticosteroids were initiated upon admission due to the marked decline in renal function and were administered empirically for suspected AIN, pending biopsy confirmation.

To better understand the pathogenesis underlying this presentation, it is important to consider previously described mechanisms of AKI in MPNs.. Patients with hematologic malignancies, including MPNs, are particularly susceptible to AKI due to glomerular endothelial damage, TMA, and direct infiltration of hematopoietic cells into the renal parenchyma [11]. These processes may impair both glomerular and tubular function, leading to abrupt renal decompensation. This framework provides a plausible explanation for the presence of ATN in our patient, superimposed on chronic histological damage [11].

Consistent with these pathological features, the RB in our patient demonstrated histological features consistent with ET-associated nephropathy, including glomerulomegaly, microaneurysms, and ATN. MPN-related glomerulopathy, as described by Said et al. [2], encompasses mesangial sclerosis, glomerulomegaly, and megakaryocytic infiltration. Notably, the presence of intraluminal megakaryocytes within glomerular and peritubular capillaries in our case suggests a potential mechanism of microvascular obstruction contributing to renal injury. This finding reinforces the association between thrombocytosis and histopathological changes such as mesangial sclerosis and chronic TMA, with circulating hematopoietic cells possibly playing a role in the progressive decline of renal function [2].

Taken together, these findings underscore the clinical relevance of renal dysfunction in MPNs. In a large multicenter study, Gecht et al. demonstrated that renal impairment in patients with MPNs, including those with ET, is independently associated with an increased risk of thrombotic events and more advanced disease severity. Specifically, patients with an eGFR below 60 mL/min/1.73 m^2^ showed a significantly higher incidence of thrombosis and longer MPN duration, highlighting the prognostic value of renal dysfunction in this population [12].

Complementing these findings, d’Izarny-Gargas et al. conducted a large biopsy-based study and identified a consistent histopathologic pattern of glomerular and vascular injury in MPNs. This included mesangial sclerosis, glomerulomegaly with megakaryocyte infiltration, chronic TMA, endothelial hyperplasia, and peritubular capillary involvement by hematopoietic cells—reinforcing the recognition of MPN-related glomerulopathy as a distinct pathological entity [13]. These lesions were significantly associated with myelofibrosis and poorer renal outcomes.

In line with this evidence, a recent systematic study by Büttner-Herold et al. examined 29 renal biopsies from patients with MPN or MDS/MPN overlap and found a high prevalence of glomerular injury, including segmental and global glomerulosclerosis, mesangial sclerosis, endothelial swelling, TMA, and intracapillary platelet aggregates. These findings support the role of chronic endothelial injury and hematopoietic cell-mediated mechanisms in the pathogenesis of MPN-associated nephropathy [14].

Further supporting this concept. glomerular capillary aggregation of hematopoietic cells has also been reported to promote endothelial damage and precipitate TMA [3], while thrombocytosis in ET and polycythemia vera (PV) is known to increase thrombotic risk and contribute to renal injury [4]. Similar glomerular changes—including glomerulomegaly, microaneurysms, mesangial proliferation, and negative immunofluorescence findings—have been described in cases of MPN-associated glomerulopathy [2]. This pathological entity is characterized by endothelial activation, microangiopathy, and the release of proinflammatory mediators, all of which may contribute to progressive renal dysfunction, manifesting as AKI or exacerbation of preexisting CKD. Renal involvement has been reported in approximately 20–30% of MPN patients, particularly in those progressing to myelofibrosis [2,15]. The underlying pathophysiology appears multifactorial, involving both direct hematopoietic infiltration and endothelial injury mediated by platelet activation and inflammatory cytokines. In our case, the identification of intraluminal megakaryocytes strongly supports the hypothesis that extreme thrombocytosis can impair renal perfusion by inducing microvascular damage.

From a therapeutic standpoint, a clear correlation was observed between increasing platelet count and deterioration in renal function, reinforcing the need for close monitoring and individualized cytoreductive adjustment. Hydroxyurea has proven effective in controlling thrombocytosis in patients with renal dysfunction while minimizing its impact on glomerular function. A phase 3 trial comparing hydroxyurea with anagrelide demonstrated that hydroxyurea more effectively reduced arterial thrombosis, bleeding events, and fibrotic progression [16]. However, its efficacy in preventing progression to ESRD remains uncertain. In this context, some studies suggest that tighter control of thrombocytosis could reduce thrombotic complications and improve renal outcomes in ESRD [5]. Although few studies have explored the correlation between thrombocytosis and renal function decline, in this case, a clear pattern of renal deterioration was observed with rising platelet counts. Renal involvement in ET and other MPNs may represent a distinct clinical subphenotype with worse long-term renal outcomes, warranting further investigation into its underlying mechanisms and optimal therapeutic strategies.

An additional consideration in the management of these patients is the need for anticoagulation to prevent major thrombotic events. The combined use of hydroxyurea and anticoagulation in patients with CKD must be carefully evaluated, balancing bleeding risk against thrombotic protection. Our patient was classified as having intermediate thrombotic risk (age > 60 years, JAK2-negative, no history of thrombosis). Although the indication for anticoagulation in ET is not well defined in this setting, it should be individualized based on comorbid cardiovascular risk factors. In this case, the patient had a known history of NVAF and was already receiving oral anticoagulation with acenocoumarol^®^, which was maintained throughout hospitalization [17].

In line with this risk–benefit assessment, anticoagulation with acenocoumarol^®^ was continued due to the presence of NVAF, while antiplatelet therapy was deliberately withheld. This decision was based on the high hemorrhagic risk associated with extreme thrombocytosis and underlying advanced CKD. Although some studies support the use of low-dose aspirin in patients with MPNs to reduce thrombotic risk, platelet counts exceeding 1,000,000/μL are associated with acquired von Willebrand syndrome and platelet dysfunction, significantly increasing the risk of severe bleeding. Current guidelines recommend caution—or even avoidance—of antiplatelet therapy in this setting. Therefore, an individualized approach was adopted, prioritizing a careful balance between thrombotic and hemorrhagic risk [7]. Of note, no randomized clinical trial has directly evaluated the impact of adding a cytoreductive agent to aspirin, leaving a gap in the evidence regarding optimal therapeutic strategies in this context [17]. Recent studies have proposed the use of direct oral anticoagulants in patients with ET and renal involvement, but evidence remains limited, and prospective trials are needed to define the most effective and safest approach [6].

To further evaluate disease progression, a BMB was performed to exclude transformation of ET into myelofibrosis, which could potentially explain the deterioration in renal function. The BMB revealed moderate reinforcement of the reticulin network (MF-2), indicating a moderate degree of marrow fibrosis. The absence of an increased CD34+ blast population suggested that, although there were signs of disease progression, transformation to advanced myelofibrosis had not yet occurred. A sparse population of CD3+ interstitial lymphocytes was noted, but did not support a significant inflammatory component. Although the patient has shown a favorable clinical response to therapeutic adjustment thus far, close follow-up is warranted to monitor for potential complications, including progression to myelofibrosis and further renal decline.

To contextualize our findings, we conducted a comprehensive literature review to assess the incidence and histopathological spectrum of renal involvement in patients with MPNs. While CKD is increasingly recognized in this setting, biopsy-confirmed AKI remains an exceedingly rare complication. Only isolated case reports and small series describe AKI associated with MPNs, and even fewer provide histologic confirmation. To address this gap, we have updated the manuscript to include a detailed summary table of renal biopsy findings reported in the literature (Table 2). These additions aim to underscore the novelty of this case and highlight the clinical relevance of ET-associated AKI as a distinct and underrecognized entity.

## 4. Conclusions

In conclusion, this case emphasizes the importance of close monitoring of renal function in patients with myeloproliferative neoplasms, dynamic adjustment of cytoreductive therapy, and the need for further research to elucidate the impact of thrombocytosis on the progression of renal failure. Renal involvement in ET patients is poorly documented in the literature, but histological findings suggest that microvascular dysfunction and hematopoietic infiltration may play a key role in the pathogenesis of renal damage.

## Figures and Tables

**Table 1:** Analytical parameters upon admission.

		Normal Values-Unit
WBC	4.13	4–10 × 10^3^/µL
Hb	11.8	12–16 g/dL
Platelet count (Plt)	1350	150–450 × 10^3^/µL
leukocytes	12.8	3.8–11 × 10^3^/µL
Mean Platelet Volume (MPV)	9.7	7–10.5 fL
Reticulocyte count	2.7	2–4%
LDH	694	120–246 U/L
Coombs test	Negative	NA
Total bilirubin	0.7	0.2–1.3 mg/dL
Indirect bilirubin	0.3	0–1.1 mg/dL
Total protein (TP)	6.1	6.4–8.7 g/dL
Serum albumin (Alb)	3.7	3–5.5 g/dL
GOT	15	5–32 IU/L
GPT	9	5–33 IU/L
CK	<15	46–171 IU/L
PCR	0.72	<0.5 mg/dL
NT-proBNP	>35,000	<300 pg/mL
Serum Urea (sU)	210	17–60 mg/dL
Serum Creatinine (sCr)	7.2	0.7–1.2 mg/dL
Serum Sodium (Na)	141	135–145 mmol/L
Serum potasium (K)	6.3	3.5–5.5 mmol/L
Serum Clorum (Cl)	107	95–110 mmol/L
Serum Magnesium	2.25	0.1–0.5 mg/dL
HbsAg	Negative	NA
HCV-Ab	Negative	NA
HIV	Negative	NA
C3 nephritic factor (C3NF)	Negative	Ratio > 1.022
C3	122	90–180 mg/dL
C4	26	10–40 mg/dL
RF	Negative	<15 IU/mL
ANA, anti-ds-DNA, ANCA, and cryoglobulin	Negative	NA
Anti-GBM	Negative	<1 IU
Anti-PLA2R Ab (ELISA)	Negative	NA
IgG	82	800–1600 mg/dL
IgA	140	70–400 mg/dL
IgM	982	90–180 mg/dL
β2-microglobulin	0.20	0–20 mg/dL
24 h urine total protein excrection	0.78	<0.15 g/24 h
Urine red blood cells	25–30	RBC/HPF
SPEP/SIFE	Polyclonal	NA
UPEP/UIFE	Negative	NA
FLC κ	9	4.90–13.70 mg/L
FLC λ	10	7.60–19.50 mg/L
FLC κ/λ	0.9	neg
INR	2.3	0.79–1.2

NA: Not applicable; WBC: White blood cells; GOT: Glutamate-oxaloacetate transaminase; GPT: Glutamate-pyruvate transaminase; Na: Serum sodium; K: Serum potassium; Cl: Serum chloride; C3: Complement component 3; C4: Complement component 4; RF: Rheumatoid factor; ANA: Antinuclear antibody; ANCA: Anti-neutrophil cytoplasmic antibody; SPEP: Serum protein electrophoresis; SIFE: Serum immunofixation electrophoresis; UPEP/UIFE: Urine protein electrophoresis/Urine immunofixation electrophoresis; FLC: Free light chains; κ: Kappa; λ: Lambda; RBC/HPF: Red blood cells per high-power field.

**Table 2:** Summary of reported renal histopathological findings in patients with myeloproliferative neoplasms (MPNs).

Reference	MPN Type(s)	No. of Cases	Renal Findings	AKI Presence	Outcome
Okuyama et al., 2007 [18]	PV	1	FSGS on biopsy; persistent proteinuria	Not reported	Worsening renal function
Said et al., 2011 [2]	PMF (8), ET (1), PV (1), CML (1)	11	Mesangial sclerosis, segmental sclerosis, chronic TMA, megakaryocyte infiltration	No	ESRD in 4 cases
Paule et al., 2013 [3]	Various MPNs	Not specified	Proteinuria, hematuria, FSGS, TMA	Not always reported	CKD progression
Lucijanic et al., 2022 [15]	MPNs	Not specified	Mesangial expansion, TMA, glomerulosclerosis	Sometimes	CKD or progressive decline
Bridoux et al., 2021 [11]	MPNs, hematologic malignancies	Not specified	Endothelial damage, tubular necrosis, hematopoietic infiltration	Yes	AKI linked to infiltration/TMA
Gecht et al., 2021 [12]	ET, PV, PMF (*n* = 685)	Registry study	Reduced eGFR, no biopsy	Not specified	Worse thrombosis and prognosis
d’Izarny-Gargas et al., 2023 [13]	ET, PV, PMF	50+	FSGS, MPGN, mesangial sclerosis, TMA, endotheliosis	Rare cases	Variable; chronic lesions dominate
Büttner-Herold et al., 2023 [14]	MPN, MDS/MPN	7	FSGS-like lesions, endothelial proliferation, TMA	Some	CKD common; some ESRD
Jain et al., 2025 [19]	ET	1	Renal artery thrombosis, endothelial injury (biopsy-confirmed)	Yes	Severe AKI with renovascular hypertension

Abbreviations: AKI: acute kidney injury; CKD: chronic kidney disease; ET: essential thrombocythemia; PV: polycythemia vera; PMF: primary myelofibrosis; CML: chronic myeloid leukemia; MPN: myeloproliferative neoplasm; TMA: thrombotic microangiopathy; FSGS: focal segmental glomerulosclerosis; MPGN: membranoproliferative glomerulonephritis; eGFR: estimated glomerular filtration rate; ESRD: end-stage renal disease.

## Data Availability

No new data were created or analyzed in this study. The data used to support the findings of this study are available from the corresponding author on request (contact J.C.D.L.F., jflomer@mde.es).
